# FMF in heterozygotes: are we able to accurately diagnose the disease in very young children?

**DOI:** 10.1186/1546-0096-9-S1-P17

**Published:** 2011-09-14

**Authors:** Véronique Hentgen, Katia Stankovic Stojanovic, Gilles Grateau, Serge Amselem, Isabelle Jeru

**Affiliations:** 1 French Reference Centre for Auto-Inflammatory Diseases (CeRéMAI), Versailles Hospital, 177, rue de Versailles, 78150 Le Chesnay Cedex, France

## Background

Reports of heterozygous carriers of a *MEFV* mutation presenting a FMF phenotype are increasing, but no data is available on the outcome of FMF heterozygous children.

## Aim

To assess the relevancy of clinical diagnosis of FMF in heterozygous children before the age of 6.

## Methods

We performed a retrospective single-centre study of 29 patients diagnosed with FMF before the age of 6, who had only 1 mutation in the *MEFV* gene, compared to a group of 26 homozygous or compound heterozygous patients in whom the diagnosis of FMF was also made during early childhood.

## Results

Presenting signs in heterozygous children did not differ from homozygous or compound heterozygous patients. Initial response to colchicine was identical in the two groups. During follow-up heterozygous patients were more likely to have a milder course of the disease. After puberty clinical signs of FMF totally disappeared in 6/11 heterozygous patients. In these 6 patients, colchicine could be withdrawn without recurrence of symptoms or rise of inflammatory markers. If applied after puberty, clinical diagnostic criteria sets were no longer positive in these 6 patients, whereas the same criteria applied retrospectively during early childhood concluded to FMF.

## Conclusion

Our study suggests that the diagnosis of FMF in very young heterozygous children should be cautious. Heterozygous children can present with an FMF-like disease during early childhood that may disappear with age, while others will suffer lifelong from their disease. Only a careful follow-up of FMF heterozygotes allows an accurate diagnosis over time.

